# Lavage, Simethicone, and Prokinetics—What to Swallow with a Video Capsule

**DOI:** 10.3390/diagnostics11091711

**Published:** 2021-09-18

**Authors:** Martin Keuchel, Niehls Kurniawan, Marc Bota, Peter Baltes

**Affiliations:** Agaplesion Bethesda Krankenhaus Bergedorf, Academic Teaching Hospital of the University of Hamburg, Clinic for Internal Medicine, 21029 Hamburg, Germany; kurniawan@bkb.info (N.K.); bota@bkb.info (M.B.); baltes@bkb.info (P.B.)

**Keywords:** capsule endoscopy, oesophagus, stomach, small bowel, colon, pan-intestinal, cleansing, lavage, PEG, NaP, prokinetics, simethicone, visualization of mucosa, diagnostic yield

## Abstract

The development of new capsules now allows endoscopic diagnosis in all segments of the gastrointestinal tract and comes with new needs for differentiated preparation regimens. Although the literature is steadily increasing, the results of the conducted trials on preparation are sometimes conflicting. The ingestion of simethicone before gastric and small bowel capsule endoscopy for prevention of air bubbles is established. The value of a lavage before small bowel capsule endoscopy (SBCE) is recommended, although not supported by all studies. Ingestion in the morning before the procedure seems useful for the improvement of mucosa visualization. Lavage after swallowing of the capsule seems to improve image quality, and in some studies also diagnostic yield. Prokinetics has been used with first generation capsules to shorten gastric transit time and increase the rate of complete small bowel visualization. With the massively prolonged battery capacity of the new generation small bowel capsules, prokinetics are only necessary in significantly delayed gastric emptying as documented by a real-time viewer. Lavage is crucial for an effective colon capsule or pan-intestinal capsule endoscopy. Mainly high or low volume polyethylene glycol (PEG) is used. Apart from achieving optimal cleanliness, propulsion of the capsule by ingested boosts is required to obtain a complete passage through the colon within the battery lifetime. Boosts with low volume sodium picosulfate (NaP) or diatrizoate (gastrografin) seem most effective, but potentially have more side effects than PEG. Future research is needed for more patient friendly but effective preparations, especially for colon capsule and pan-intestinal capsule endoscopy.

## 1. Introduction

Since the advent of video capsule endoscopy in 2000, a steady development of hardware, software, procedure, and indication took place. Starting with a small bowel capsule in fasted patients having suspected mid gastro-intestinal (GI) bleeding, indication was expanded to Crohn’s disease, complicated sprue, polyposis, and tumors of the small bowel.

Non-invasive video capsule endoscopy is limited by its passive propulsion and inability to clean or distend the GI lumen. Various types of bowel preparation have been implemented to increase mucosa visualization and prokinetics were applied to improve complete visualization of the small bowel. Completeness and cleanliness have been identified, amongst others, as quality parameters by the European Society of Gastrointestinal Endoscopy (ESGE) [1], stressing the need for proper bowel preparation. On the other hand, as a non-invasive procedure, capsule endoscopy aims to minimize patients’ discomfort, e.g., by avoiding unnecessary lavage. New generations of capsules with longer battery time and higher resolution have shifted the focus from incomplete small bowel visualization to optimal cleanliness. Although many studies have been performed in this area over the last two decades, the results are as-yet often conflicting.

Introducing new types of capsules for the esophagus, stomach, colon, or entire GI tract has created very different needs for preparation of the GI tract to allow best visualization of the mucosa of each organ. The distension of the stomach in magnetically controlled capsule endoscopy or the propulsion of a colon capsule to and through the complete colon are new challenges.

This paper reviews the aspects of preparation for the different capsule endoscopies of esophagus, stomach, small bowel, and colon using lavage, anti-foaming agents, and prokinetic drugs. Furthermore, some of the scores used for assessment of their effect are described.

## 2. Oesophageal Capsule Endoscopy

For this investigation, the required pre-interventional measures are the least invasive. Two hours of fasting, followed by drinking 100 mL of water in the upright position has been suggested to clean saliva from the oesophagus [2]. The capsule is propelled by the ingestion of 15 mL water from a syringe every 30 s until the capsule has reached the stomach [3]. The cleansing level on the Z line area in PillCam Eso2 has been scored as: 0 (no), 1 (minor) or 2 (major) interference by bubbles/saliva [4].

## 3. Gastric Capsule Endoscopy–Magnetically Controlled Capsule Endoscopy (MCE)

### 3.1. Procedure of Gastric Capsule Endoscopy

Soft food the day before the examination, fasting for least 8 h overnight and no oral medication or coloured fluids for at least 12 h have been recommended before gastric capsule endoscopy. One litre of water is ingested 10 min before swallowing the capsule to distend the large gastric lumen and is repeated during the examination if needed. When combining gastric and small bowel investigation, lavage prior to examination is recommended [5]. In a randomized trial including 120 patients, adding simethicone to water ingestion before MCE improved the mean total cleanliness scores from 15.83 ± 2.41 to 21.35 ± 1.23 and mean total visualization scores from 10.75 ± 2.02 to 15.20 ± 1.32 (*p* < 0.0001, each). However, adding pronase to water and simethicone had no further effect [6].

Repetitive position changes for 15 min after the ingestion of dimethicone significantly improved the number of patients with acceptable gastric cleanliness scores from 72.5% to 100% (*p* < 0.001) in a randomized trial. However, diagnostic yield was not different in both groups [7].

Preceding lavage is only necessary if gastric capsule endoscopy is combined with a small bowel procedure once the capsule has been directed into the descending duodenum.

As steering of the capsule within the stomach is enabled by an external magnet, either handheld or by dedicated computer-controlled magnet, prokinetics are not applied.

### 3.2. Scores for Gastric Capsule Endoscopy

A **gastric cleanliness score** based on six primary anatomical landmarks of the stomach (cardia, fundus, body, angulus, antrum, and pylorus) using a four-point grading scale has been proposed. The grading is as follows: excellent (no more than small bits of adherent mucus and foam: score 4); good (small amount of mucus and foam, but not enough to interfere with the examination: score 3); fair (considerable amount of mucus or foam present precluding a completely reliable examination: score 2); and poor (large amount of mucus or foam residue: score 1). Additionally, a 3-point grading scale for mucosal visualization is calculated from each of the six anatomical landmarks as mentioned above as good (>90% of the mucosa observed: score 3), fair (70–90% of the mucosa observed: score 2) and poor (<70% of the mucosa observed: score 1). The scores for total gastric cleanliness and total mucosal visualization each are calculated as the sum of individual scores of the six anatomical landmarks [6].

## 4. Small Bowel Capsule Endoscopy (SBCE)

Optimal cleanliness is crucial for reliable diagnosis with SBCE. For instance, poor visual quality was associated with lower diagnostic yield in small bowel malignancy [8]. ESGE has defined a target of >95% SBCE procedures with adequate small bowel visualization as key performance parameter [1]. To achieve this goal, anti-foaming agents are applied to minimize air bubbles, lavage to reduce debris. Another key performance parameter is complete visualization of the small bowel. Prokinetics may be used to shorten gastric and/or small bowel transit to increase the small bowel completion rate (target > 80%).

### 4.1. Anti-Foaming Agents

**Figure 1:**
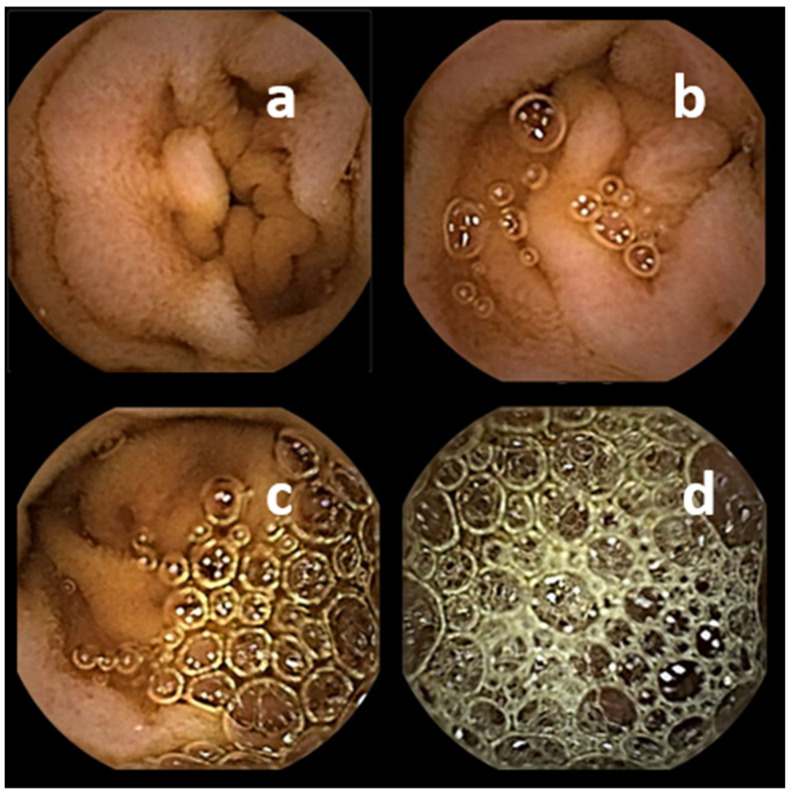
SBCE: Abundance of air bubbles: (**a**) none, (**b**) minimal, (**c**) mild, (**d**) severe.

Air bubbles are a significant obstacle for the adequate visualization of gastrointestinal mucosa. Several studies demonstrated improved visualization of small bowel mucosa after application of **simethicone** before swallowing the capsule. A meta-analysis found an Odds Ratio (OR) for adequate mucosa visualization of 2.84 (95% Confidence Interval (CI) 1.74–4.65) after application of simethicone before SBCE [9]. Doses between 80 mg to 300 mg of this antifoaming agent have been used. In consequence, simethicone before SBCE has been recommended by European (ESGE) [10], Canadian [11], and German [12] guidelines. However, a recent randomized trial found no improvement of mucosal visualization by high-dose simethicone (1125 mg) compared to standard dose (300 mg) additionally to PEG based lavage the day before SBCE. [13]. A score with four grades has been used for human operator assessment: no intraluminal gas; no/moderate/severe limitation of visibility by intraluminal foam/gas bubbles [14] (Figure 1). Additionally, a computer generated score based on different algorithms from still SBCE images aims to differentiate images with less or more than 10% abundance of bubbles [15].

### 4.2. Lavage for SBCE

**Figure 2:**
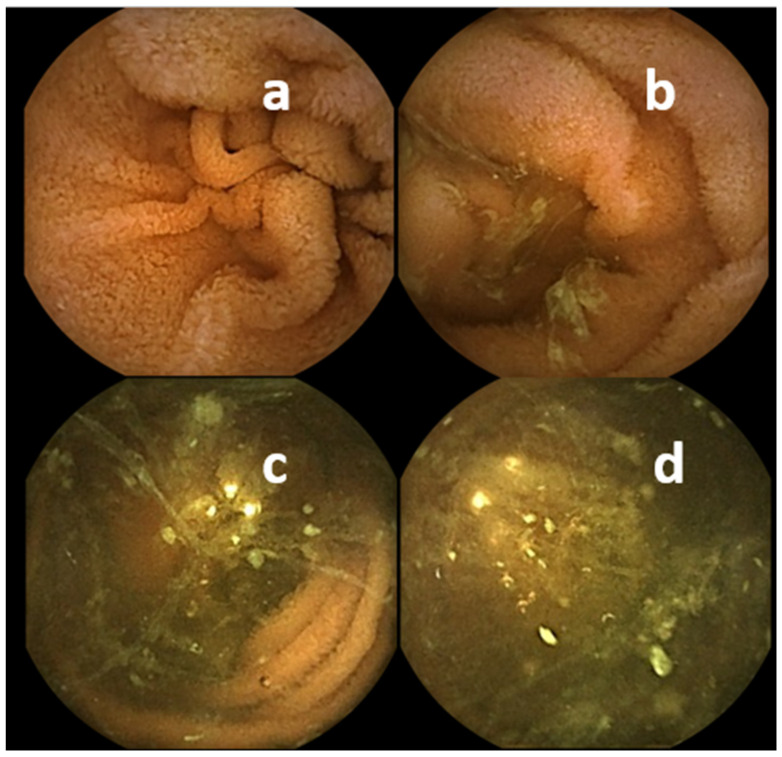
Mucosa visualization in SBCE: (**a**) perfect, (**b**) good, (**c**) fair, (**d**) poor.

Use of lavage before SBCE is recommended by international guidelines of ESGE [10], and national guidelines in Korea [16], Canada [11], and Germany [12], while ASGE technical report mentions clear liquids as standard, but also mentions studies that show a benefit of lavage [17]. However, there are various regimens for lavage with respect to the type, volume, and timing of lavage. Furthermore, scores assessing the effect of lavage differ between studies and the results between investigators, and the conclusions drawn in several meta-analyses from these studies vary accordingly. While some producers of video capsule systems only recommend an 8 to 12 h fasting period, clinical practice shows that debris can significantly hamper proper visualization of small bowel mucosa (Figure 2).

#### 4.2.1. Benefit of Lavage before SBCE

A growing number of trials has investigated the effect of bowel prep on the visualization of small bowel mucosa during capsule endoscopy, increasingly with prospective randomized design. There are several meta-analyses on these studies (Table 1). The high number of such meta-analyses per se is a hint about the existence of conflicting results. Depending on the inclusion criteria of the studies, their endpoints, different interventions, and different assessment scores, conclusions vary.

The first meta-analysis by Niv et al., 2008 [18] found an improved visualization of small bowel mucosa after prep. However, besides polyethylene glycol (PEG) and sodium picosulfat (NaP) as lavage, simethicone as anti-foaming agents was also included as an intervention. Diagnostic yield was not studied. Following meta-analyses by Rokkas et al., 2009 [19], Belsey et al., 2012 [20], and Kotwal 2015 [21] showed a significantly higher proportion of patients with good or excellent cleanliness after lavage with PEG or NaP (OR 2.13–3.13). Furthermore, diagnostic yield was significantly higher with lavage in all three meta-analyses (OR 1.77–1.88). Two more meta-analyses confirmed slightly better mucosa visualization with PEG [22] or either PEG, NaP or citrate before SBCE [23], but no improvement in diagnostic yield.

Finally, a last meta-analysis by Gkolfakis et al., 2018 questioned the benefit of lavage before SBCE, as only an improvement of visualization with NaP was demonstrated, but not for PEG or magnesium citrate [24]. The methodology issues of the meta-analysis are the exclusion of studies not presenting the outcome in a dichotomic manner as adequate or inadequate for the entire capsule video but providing scales for cleanliness and assessing different small bowel segments separately (see below).

#### 4.2.2. Type of Lavage

Most studies compared PEG or NaP with clear liquids beginning the day before SBCE. Some of the meta-analyses included subgroups comparing PEG and NaP with ambiguous results. Two found a benefit of PEG over NaP [20,21], while in three others NaP was superior to PEG [19,23,24]. Due to less potential side effects, especially renal, ESGE recommendations prefer PEG [10]. A European consensus group recommended not using NaP in patients with chronic kidney disease, pre-existing electrolyte disturbances, congestive heart failure, cirrhosis or a history of hypertension [25]. Furthermore, to avoid small artificial small bowel lesions potentially caused by NaP itself, it should be avoided [26].

Other substances such as mannitol [27], citrate and low volume PEG/ascorbic acid (Moviprep) have been used successfully [28,29]. Very low PEG/ascorbic acid lavage (Plenvu) has also been used successfully in clinical practice, but not studied in controlled trials. Finally, 4 L of clear liquids before SBCE were not inferior in a randomized controlled trial with 245 patients to 2 L or 4 L of PEG, but associated with fewer side effects [30]. However, all patients were on clear liquids the entire day before SBCE; and PEG or 4 L clear liquids were all ingested the day before and not in the morning of the procedure.

#### 4.2.3. Volume of Lavage

Apart from various types of lavage, different volumes have been used. A cumulative ranking network meta-analysis of several studies found 2 L of PEG superior to 1 L or 4 L [31]. Accordingly, a dose of 2 L of PEG is recommended in the technical report of ESGE [10]. A recent randomized trial with five arms compared no PEG, 1 L, or 2 L of PEG 12 h before capsule endoscopy, and 1 L or 2 L of PEG 4 h before SBCE. The best quality of images and best acceptance by patients was found with 1 L of PEG 4 h before SBCE. Furthermore, diagnostic yield was significantly higher with PEG than without (29% vs. 11%; *p* < 0.005) [32].

In 198 children, a five-arm randomized controlled trial investigated 12 h of clear liquids, 376 mg simethicone, 50 mL/kg PEG, and 25 mL/kg PEG with and without simethicone. Best visualization was obtained in the group with 25 mL/kg PEG plus 376 mg Simethicone. Diagnostic yield and tolerability were not different [33].

#### 4.2.4. Timing of Lavage

Large amounts of gastric and biliary juice are also produced and excreted into the small bowel overnight. Hence, a split dose regimen with lavage the day before SBCE and additionally a portion in the morning before the investigation seems useful for washing out dark fluids. Improved mucosal visualization could be obtained when 1 L of PEG each was ingested in the evening and 4 h before SBCE compared with 2 L PEG only in the evening [34].

**Figure 3:**
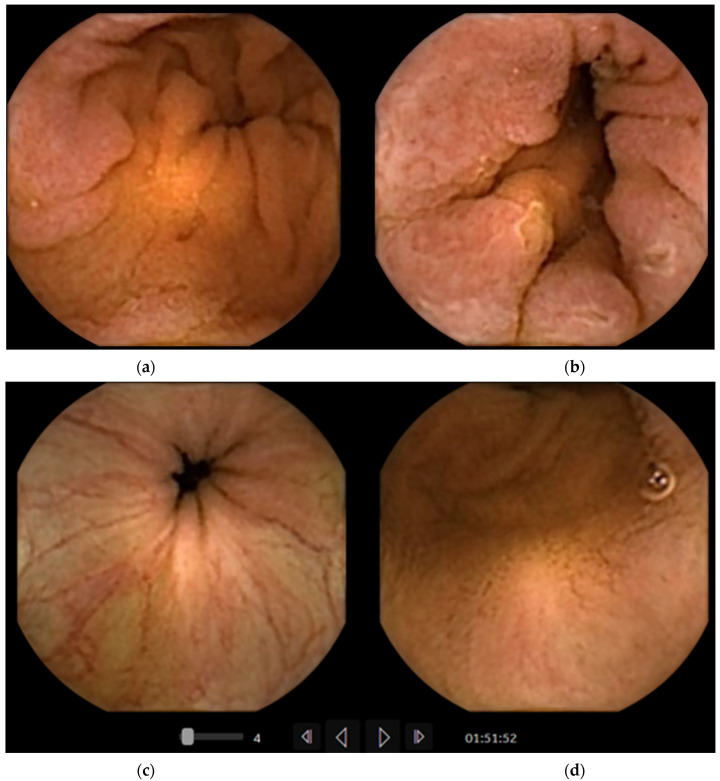
Perfect mucosa visualization in the terminal ileum after lavage and booster. (**a**,**b**) Pan-intestinal capsule PillCam Crohn’s, b with aphthous ulcer; (**c**,**d**) normal terminal ileum in colon capsule PillCam Colon2.

Practical experience with colon and pan-intestinal capsule endoscopy also shows a clear lumen in the distal small bowel (Figure 3). This could be due to additional cleansing of the colon, thus avoiding reflux of the remnants to the ileum. However, major reason seems to be the effect of standard ingestion of lavage after the colon capsule has reached the small bowel, thus washing dark bile from the distal ileum. This concept of lavage ingestion after the colon capsule has reached the SB has also been adopted for SBCE. the time of ingestion had either been chosen by expected mean gastric transit time (GTT) or by documentation of small bowel mucosa at real time viewing during the procedure. When drinking large volumes beforehand, a delay in gastric motility with further delay in passage of the capsule to the duodenum is anticipated. Patients drinking 500 mL of PEG between 30 and 60 min after ingestion of the capsule had significantly better visualization of mucosa in the distal small bowel compared to those who had ingested only clear liquids since the preceding day [35,36,37].

Adler and coworkers found a better visualization of distal SB mucosa with Picolax (NaP and citrate) 1 h after ingesting the capsule compared with PEG the evening before SBCE [38]. A recent prospective trial compared 1 L of Moviprep after the capsule had entered the small bowel with Moviprep the day before. Good or excellent visualization was documented by two investigators in 79/76% compared with 45/42% after Moviprep in the evening (*p* < 0.005). The diagnostic yield for angiectasias, the most frequent finding, was also higher, with 27% vs. 10%, respectively (*p* = 0.022) [28]. Both groups stayed on clear liquids the day before SBCE and fasted overnight.

#### 4.2.5. Influence of Lavage on Small Bowel Transit

Complete visualization of the entire small bowel is a prerequisite for correct diagnostics. The first generations of capsules with limited battery lifetime had a relevant percentage of incomplete investigations. In a comparative study with two different capsules swallowed in parallel, all four patients in whom PillCam had missed a relevant lesion with bleeding potential, small bowel visualization was only complete with EndoCapsule but not with PillCam.

Hence, the use of prokinetics and influence of lavage on SB transit were topic of several studies, aiming to increase the SB completion rate [39]. However, studies could not demonstrate a higher rate of compete SB visualization following lavage. As lavage was ingested the day before investigation this is not unexpected.

On the other hand, 500 mL of PEG 30 min after ingestion of the capsule did not only improve image quality, especially in the distal small bowel, but also increased the rate of capsules reaching the cecum within battery lifetime [37]. Five hundred millilitres PEG swallowed 60 min after the capsule (optionally with metoclopramide if the real time viewer found the capsule still in the stomach) shortened mean small bowel transit by 43 min. Additionally, visualization of the distal small bowel was significantly better [35]. In another study including ingestion of 500 mL PEG 30–120 min after swallowing the capsule, visualization was improved and SB transit shortened, but the completion rate was not increased [36].

Studies using newest PillCamSB3 generation with longer working capacity could not find a higher completion rate for the small bowel when lavage was ingested after the capsule [28,38]. However, the completion rate in the entire study population was already 94% [28]. The longer battery time of newest generation of all capsule platforms provide a high rate of complete small bowel diagnostics, reducing the need to speed up SBTT.

Furthermore, concerns have arisene that increasing the SB passage and speed by delaying lavage until the capsule has entered the small bowel might reduce the diagnostic yield by missing some lesions. This problem could be tackled by increasing the image capture rate of capsules, such as an adaptive frame rate of up to 6 frames/s in PillCamSB3. Using PillCam SB3, Adler et al. observed a shorter small bowel transit with Picolax (NaP and magnesium citrate) after capsule entry into small bowel compared with PEG on the evening before (194 min vs. 248 min; *p* < 0.01). Although diagnostic yield was not different there was a trend towards lower DY in the group with faster passage 6/18 (33%) vs. 4/18 (22%) However, another study detected more angiectasias when Moviprep lavage was drunk after swallowing the capsule compared to the eve of SBCE. The transit times were not different between both groups [28].

### 4.3. Scores for Small Bowel Mucosal Visualisation

A variety of scores has been developed to assess quality of visualisation of small bowel mucosa. A comprehensive overview has been published recently [40]. In clinical practice only human operator-based scores are used, as several computer-based scores have been developed, but are not yet implemented in commercially available software [41]. Human operator-based scores are readily applicable, but subjective. **Brotz** et al. suggested and validated three scores: an overall assessment (OAA), a qualitative evaluation (QE), and a quantitative index (QI) [42]. A dichotomic overall assessment into adequate (>90% of mucosa visualized) or inadequate (<90% of mucosa visualized; study should be repeated) is the simplest one. A somehow more detailed assessment (Figure 2) further specifies adequate into excellent (absent or minimal impairment) and good (mild impairment) by fluid and debris abundance, bubble abundance, bile/chyme staining, and brightness reduction. Accordingly, inadequate comprises fair (moderate impairment, 80–89% of mucosa visualized), and poor (severe impairment, <80% mucosa visualized). Based on these parameters of impairment and the percentage of visualized small bowel mucosa, a qualitative index was built by adding five scores 0, 1, or 2 for each of the five parameters.

However, in a recent French multicentre study with 3 experts reading 155 videos, agreement was poor. Intraobserver reproducibility was fair to moderate, with kappa coefficients between 0.37 and 0.46 for QI, 0.41 and 0.51 for QE, 0.41 and 0.50 for OAA. Inter-observer reproducibility was fair to substantial according to kappa coefficients between experts varying from 0.40 to 0.64, 0.29 to 0.65, and 0.52 to 0.71, for QI, QE and OAA, respectively [43].

A **Korea–Canada (KODA) score** [23] has been proposed for future use in studies. Sequential images selected in 5 min intervals from the SBCE videos were rated on a scale between 0–3 based on the amount of visualized mucosa and the degree of obstruction. Twenty-five SBCE videos with 1233 images were rated by 20 readers twice in a 4 week interval. Intraclass correlation coefficients for inter-rater (ICC 0.81, 95% CI 0.70–0.87) and intraobserver (ICC 0.92, 95% CI 0.87–0.94) reliability were almost perfect. Some studies applied more differentiated classifications for assessing small bowel cleanliness. Hosono et al. [35] classified mucosa visualization gradually in steps of 20% separately for each 10% of small bowel transit. A ‘visibility score’ based on percentage of visualized mucosa was classified as follows: 1 (<25% mucosa visualized); 2 (25–49%); 3 (50–74%); 4 (75–89%); 5 (>90%) [36]. Van Tuyl et al. recorded the percentage of patients with adequate cleanliness for all four small bowel quartiles [44]. All three studies mentioned above found a decrease in visualization in distal small bowel areas, but also a significant improvement by lavage with PEG before SBCE.

The meta-analysis by Gkolfakis et al. [24], questioning the benefit of lavage before SBCE, however, did not include the three studies with a significant effect of PEG predominately in the distal small bowel because they used differentiated scores instead of a dichotomic overall assessment (adequate/inadequate). Thus, a bias in this meta-analysis cannot be excluded.

These results demonstrate the need for objective and easy to use assessment. Computer generated scores have been proposed but are not yet incorporated into commercially available reading software. Scores based on red/green ratio of entire PillCam videos have been suggested [45,46], and adapted for the MiroCam system [47]. A combination of colour, brightness, and abundance of bubbles has been included in a computer-generated score from 0 to 10 based on still images. A cut-off of 7 had a sensitivity of 90.0% [95% CI 84.1–95.9], and a specificity 87.7% (95% CI 81.3–94.2)) [48].

### 4.4. Prokinetics

Different medications affecting GI motility have been studied for their effect on the completion rate of SBCE. The first drug investigated was the antidopaminergic 5-Hydroxytryptamine-3 (5-HT 3) agonist **metoclopramide (MCP)**. An increased rate of complete small bowel investigation after 10 mg of MCP had been described for PillCam SB1 in a group of 67 patients compared to a historic cohort of 80 patients (97% vs. (OR 10.3; 95% CI 2.32, 93.55), probably due to accelerated gastric transit (47.9 ± 9.0 min vs. 30.8 ± 7.5 min; *p* = 0.025 [49]. In another randomized trial with OMOM capsule, the SB completion rate was not different in spite of a shortened gastric transit with MCP injected intramuscular with capsule ingestion [50]. Likewise, in a randomized four-armed trial, 10 mg of MCP orally 10 min before ingesting PillCam SB1 additionally to simethicone and either clear liquids from the afternoon before, followed by NPO or bowel prep with magnesium citrate (Citramag) and senna did not influence the small bowel completion rate [51].

Unlike MCP, the dopamine-2 receptor antagonist **domperidone** does not cross the blood–brain barrier and is acting more specifically in the stomach. In a retrospective comparison of 31 patients receiving 20 mg of domperidone before SBCE with a historic cohort of 33 patients, astonishingly, an even prolonged oro-duodenal transit from 13 to 30 min (*p* < 0.01) was observed. There was no difference in small bowel transit [52].

A single centre trial randomized 100 children (mean age 10.1 ± 3.4 years) in a domperidone group and 100 patients to the control group (10.7 ± 3.6 years). The median GTT with 67.5 min (44.8–117.5) in the domperidone group and 80.0 min (42.0–128.0) in the control group where not significantly different (*p* = 0.49). Additionally, median small bowel transit times (SBTT) were similar with 317 min (231–436) and 323 min (225–426), respectively (*p* = 0.52). The complete examination rate of OMOM capsule was 97% in the domperidone and 98% control group, respectively (*p* = 1.00) [53].

When 200 mg of **erythromycin** (a macrolide antibiotic with motilin like activity) was given orally 1 h before SBCE, it did not influence gastric or small bowel transit [54]. However, in a sequential cohort study, 250 mg of erythromycin orally 1 h before PillCam SB1 (*n* = 239) was compared to 10 mg of oral domperidone directly before the procedure (*n* = 410). Complete small bowel visualization increased from 80% with domperidone to 86% after erythromycin (*p* = 0.03), associated with a shorter median gastric transit after erythromycin compared to domperidone (13 min versus 22 min, *p* < 0.001). Median SBTT was similar [55].

The effect of different 5-Hydroxytryptamine-4 (5-HT4) agonists—mosapride, prucalopride, and tegaserode—stimulating gastrointestinal motility on capsule transit and small bowel completion has been analysed in some studies.

Oral **mosapride** citrate (odds ratio [OR], 1.99; 95% CI 1.01 to 3.91) and GTT (OR, 2.34; 95% CI 1.13 to 4.87) were significant factors for improving the SBCE completion. Oral mosapride citrate significantly shortened the GTT and SBTT [56].

Administering 2 mg **Prucalopride** at the time of capsule ingestion was associated with a significantly shorter small bowel transit in 29 hospitalized patients—92 versus 275.5 (*p* < 0.001)—with a tendency towards higher small bowel completion rate. In a small retrospective study, prucalopride decreased median small bowel transit from 275.5 min to 92 min, *p* < 0.001 [57]. The completion rate with EndoCapsule 1—and in a later period PillCamSB3 as a secondary outcome in a retrospective study—was higher for patients who received prucalopride (90.4%, (19/21) vs. 61.9% (13/21); *p* = 0.06) [58].

**Tegaserod** (removed from market for cardio-vascular risks) in a small cohort was reported in an abstract to reduce gastric as well as small bowel transit [59].

Small bowel transit after ingesting 290 µg of **Linaclotide** (a guanylatcyclase-receptor agonist) 1 h prior to SBCE in 29 patients was similar to a historic group of 30 patients with standard PEG prep (192 min versus 202 min, respectively; *p* = 0.93) [60]. Furthermore, the proportion of patients with good or excellent image quality was similar (19/28 for linaclotide, and 18/28 for PEG). However, 2 L of PEG were ingested the day before SBCE.

In a double blinded, placebo-controlled trial with 40 healthy adults, 24 µg of **lubiprostone** (a selective type 2 chloride channel activator) given 30 min before PillCam SB1 even increased mean GTT significantly from 43 to 126 min (*p* = 0.0095). The mean SBTT was similar after lubiprostone (188 min vs. 219 min; *p* = 0.130) [61].

**Daikenchuto**, a traditional Japanese Herbal medicine, increased the rate of complete small bowel investigations from 72.7% to 96.5% [62]. However, the recorder of PillCam SB or SB2 was removed after 8 h.

In a prospective study 44 patients were randomized to no prep (only NPO 12 h before SBCE) or standard prep with 2 L of Moviprep (PEG and ascorbic acid) the night before and 5 mg of liquid metoclopramide 20 min before ingestion of the capsule. There was a higher completion rate with PillCam SB2 in the prep group, but no difference in mucosa visualization or diagnostic yield, while patient discomfort, mainly related to PEG, was higher in the prep group (62% vs. 17%, *p* < 0.01) [63].

All producers offer new generations of capsules with longer working capacity of at least 11 h, sometimes even up to 20 h. Hence, the problem of incomplete small bowel investigation is of marginal importance in the absence of gastric paresis or stenosis. To detect those patients with delayed gastric passage, the use of a real time viewer is crucial.

Application of a **real-time viewer** for identification of patients with massive delay in gastric transit has been described for PillCam [64], EndoCapsule [65,66], and OMOM capsule [50]. Tailored application of prokinetic agents in these patients could increase the small bowel completions rate. In a historic comparison, the completion rate (86% vs. 66%; *p* = 0.002) and the rate of positive findings (80% vs. 67%; *p* = 0.04) was higher in the real-time viewer group [67]. Thirty three of 100 patients needed intervention (additional water, MCP intravenously or endoscopic transport) due to detection of delayed gastric transit. Another comparison of historic groups describes the application of 10 mg domperidone orally after 1 h of capsule staying in the stomach, repeated after 30 min, and finally endoscopic transport to the duodenum if still necessary. Thus, the rate of incomplete SBCE could be reduced from 15.6% 3.7% (*p* = 0.003) [68].

In a randomized trial the completion rate was significantly higher (72.5 vs. 90.0%) when after 1 h, before swallowing additional 500 mL PEG, a real-time viewer was used and MCP given intramuscular in case the PillCam SB2 capsule was still in the stomach. Furthermore, the detection rate of lesions in the distal small bowel was higher in the real-time group than in the conventional group [35].

The general application of prokinetics is not recommended; instead, a real time viewer should be used to detect delayed gastric passage requiring prokinetics or endoscopic transport [10]. Patients at risk for delayed gastric transit seem to be bed ridden, in-patients, and intensive care patients, patients with diabetic neuropathy, severe hypothyroidism, renal insufficiency, or taking psychotropic or narcotic drugs.

## 5. Colon Capsule Endoscopy (CCE) and Pan-Intestinal Endoscopy (PCE)

**Figure 4:**
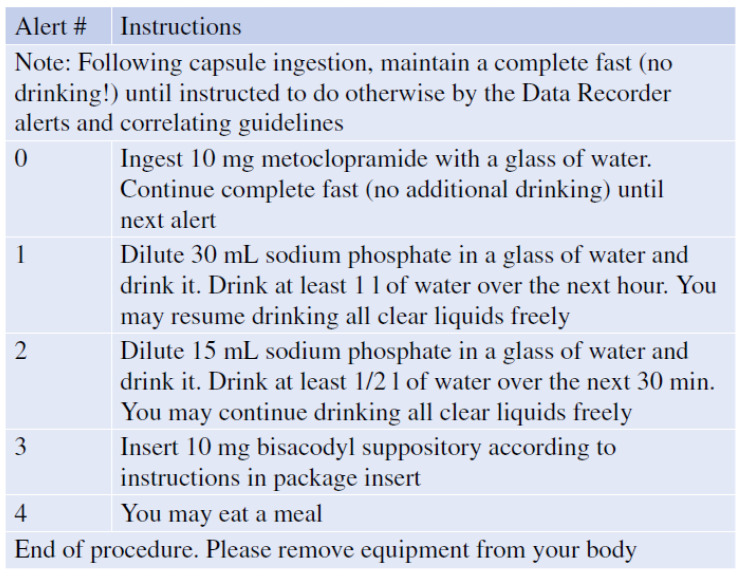
Example for programmable alerts to be shown to the patient triggered by time after capsule ingestion or by automated recognition of small bowel by PillCam Colon 2 or PillCam Crohn’s capsule system.

While there is still some debate about necessity of bowel cleansing before SBCE, for the colon there is no doubt about the need for thorough cleansing. Compared with flexible colonoscopy which allows suction and rinsing during the procedure, a rigorous cleansing regimen is mandatory before starting a CCE and during the CCE. Furthermore, the propulsion of the capsule through the colon requires some sort of boost during the procedure to ensure the complete visualization of colonic mucosa before the battery expires. The first boost is ingested once the small bowel mucosa is visualized by the real-time viewer. An alert with instructions for the patients is automatically generated by real time software algorithms (Figure 4) [69].

Hardware and procedure for CCE and PCE are very similar. Thus, the discussed aspects of prep refer to both modalities.

Details from two studies demonstrate the importance of cleanliness and completeness. Sensitivity of CCE for detection of polyps < 6 mm was 75% in patients with adequate bowel cleansing compared to only 42% in patients with inadequate prep, and for polyps > 10 mm or advanced adenomas 88% vs. 44%, respectively [70]. Sensitivity for polyps > 9 mm was 87% (95% CI 83–91%) in 253 patients with positive iFOBT. Amongst them only 126 patients had a complete CCE. In this group sensitivity was 97% (95% CI 94–100%) for >9 mm polyps [71].

### 5.1. Lavage and Boosts for CCE/PCE

An early ESGE guideline recommended following a liquid diet the day before the procedure followed by a total of 4 L of **polyethylene glycol (PEG)** before the CCE procedure. A split-dosage regimen with intake the day before and on the day of the examination was advised to increase tolerability and efficacy of the preparation, with no clear statement on the role of low-residue diet. As a boost to propel the capsule through the colon low dose, **NaP** was recommended, while practitioners were warned to be alert to potential contraindications in elderly persons, patients with bowel obstruction, active colitis, hypovolemia, kidney disease, on medications affecting renal perfusion as angiotensin-converting enzyme inhibitor [72].

Later, low volume Moviprep (**PEG plus ascorbic acid**) was applied as split-dose bowel prep before CCE and again as first and second booster resulted in complete CCE in 76%. In total, 82% of patients had an adequate overall cleanliness [73]. In another trial, excellent or good bowel cleansing could be achieved in 78% of cases with 2 L Moviprep (*n* = 28) compared to 64% of cases with 4 L PEG (*n* = 30), respectively; *p* = 0.252. However, a significantly higher excretion rate was observed: 93% with 2 L PEG + ascorbic acid vs. 70% with 4 L PEG (*p* = 0.043). In contrast to the before mentioned study, 30 mL of NaP solutions was used as first and second booster underlining its efficiency [74].

Seventy four patients underwent CCE after incomplete flexible colonoscopy with Moviprep as lavage and as booster; with NaP only as additional ‘rescue booster’ after 7 h. Bowel cleansing was adequate in 67%. CCE could complement incomplete colonoscopy in 93% but was complete in only 65% [75].

Replacing NaP boosters with PEG in a controlled trial resulted in a reduction of complete CCE from 100% to 75% (*p* = 0.02) [76].

With a more aggressive approach using prep with Senna and PEG, followed by **NaP and gastrografin** boosts, CCE achieved complete colonic evaluation in 98% in 100 patients with incomplete flexible colonoscopy. Adequate cleansing was observed in 83% (95% CI 74–90%). Although this protocol resulted in good visualization and perfect completeness rates it was accompanied by prep related adverse events in 28% (in descending order: nausea (*n* = 11), vomiting (*n* = 7), headache (*n* = 6), abdominal pain (*n* = 3), and vertigo (*n* = 1). All events were mild to moderate, resolved within the same day [77].

Following Moviprep lavage, two boosts of 50 mL gastrografin and **Magnesium citrate** (Magcorol) together with 10 mosapride with the first boost a completion rate of 97% excretion, 90% adequate cleansing could be achieved without adverse events. More than half of the patients had a previously incomplete colonoscopy [78].

A multicentre, prospective, randomized trial on CCE for colo-rectal cancer (CRC) screening compared oral **sulphate** solution (89 mL) with diatrizoate solution (gastrografin) (boost 1 = 60 mL, boost 2 = 30 mL) with a control regimen of oral sulphate solution (89 mL) alone. Adequate cleansing was not significantly different (75.9% vs. 77.3%) but complete CCE was higher with gastrografin (90.9% vs. 76.9%; *p* = 0.048). Inadequately short colon transit time of less than 40 min with the anticipated risk of missing lesions by rapid pace, occurred in 21.8% vs. 4% in the control group; *p* = 0.007) and adverse events were significantly higher with 19.4% vs. 3.4%, respectively (*p* = 0.0061) [79].

A large multicentre US trial with CCE for CRC screening in 884 asymptomatic subjects finally analysed 695 persons per protocol (79%). For bowel prep, 12 mg Senna two days before CCE, and 2 L of sulphate-free PEG each in evening and morning before CCE were ingested. As the first boost, after the capsule entered the small bowel, 6 oz of oral sulphate solution (Suprep) diluted to 16 oz with water were administered. Three ounces of oral sulphate solution diluted in water to 8 oz were swallowed as second boost three hours later if the capsule was not excreted before. Both boosts were followed by 1 L of water, each. Adequate cleansing was seen in 80% (95% CI, 76–83%). Seventy seven participants (9%) were excluded for inadequate cleansing and colon transit time <40 min. In total, 142 non-serious adverse events (AE) occurred in 101 patients (11%), of which 128 AE related to bowel preparation. All resolved within 1 month, 92% within 1 day [80].

A study with 64/70 patients scheduled for CCE showed that water intake of >12.0 mL/min during examination (OR: 46 *p* = 0.025), 95% CI: 1.63–1341] significantly influenced complete visualization of colon. Additionally, factors influencing completion of CCE within 4 h were: BMI of >25 (*p* = 0.039, OR: 13.723, 95% CI: 1.135–165.913), absence of constipation (*p* = 0.030, OR: 13.988, 95% CI: 1.287–152.047), and again water intake of >12.0 mL/min during examination (*p* = 0.004, OR: 12.028, 95% CI: 2.225–65.029) [81].

Capsule excretion within battery life was significantly higher for: age < 65 years (OR 3.00; *p* = 0.0048); male sex (OR 3.20; *p* = 0.0051) and use of **castor oil** (OR 6.29; *p* = 0.0003) [82]. However, neither chewing gum nor coffee increased the rate of complete CCE [83].

To minimize patients’ discomfort, **one-day schedules** have been proposed. In a randomized pilot trial a one-day schedule (*n* = 20) with fibre-free diet and 3 L PEG on day 0 was compared to a two-day schedule (*n* = 20) with liquid diet and 3 L PEG in the evening of day 1 and 1 L PEG in the early morning of day 0. Adding NaP in both groups overall colon cleanliness was adequate in 94% (CI 91–97%) for the one-day schedule compared with 80% (CI 72–88%) in the two-day schedule (*p* = 0.27) [84].

For the monitoring of ulcerative colitis, a one-day schedule with 500 mL PEG, followed by 250 mL of water, 2.5 h before, and 1, 3, and 6 h after ingestion of the capsule has been proposed, combined with castor oil added to the second ingestion. Excretion rate within battery lifetime was 93.9% (31/33). Acceptability of CCE-2 was superior to colonoscopy (42.4% vs. 27.3%), but not in those patients with longer colonic transit [85].

### 5.2. Prokinetics for CCE/PCE

Many protocols empirically include ingestions of **senna** (4 tablets, 12 mg) two days before the CCE procedure in the evening to stimulate bowel movements before starting the liquid diet and lavage the following days [74,77,78,80,86,87]. As an alternative, 1000 mg oral **magnesium-oxide** in the morning and evening of day 2 have been added to a protocol [71].

Considering the necessity to delay the first boost until the capsule has left the stomach, **prokinetics** are still part of actual protocols, at least in demand after 1 h. Patients were regularly given 20 mg domperidone before CCE [70,71,73,74], or on demand (if the capsule did not leave the stomach after 1 h) [86], as well as 10 mg MCP in all patients [88] or only on demand orally [80] or intravenously in saline [89]. Either 10 mg MCP or 250 mg erythromycin (if the capsule stayed for >1 h in the stomach) have been applied for CCE [79] and for PCE [90]. Furthermore, 30 mg domperidone and 10 mg mosapride together with capsule ingestion have been proposed [78]. Additionally, Mosapride 5 mg [91] or 15 mg [92]), as well as Tegaserod 10 mg [93] were used in CCE protocols. However, there are no randomized trials on this specific issue.

Finally, many protocols recommend a 10 mg **bisacodyl suppository** to stimulate evacuation of the capsule if it had not been excreted following the second boost after either 2 h [70,74,75,76,77,78,79,86,87], 2.5 h [70], or 3.5 h [73] without systematic evidence but in the absence of alternatives. Another study included Bisacodyl supplements on the day before a one-day procedure [84].

### 5.3. CCE Cleansing Score

**Figure 5:**
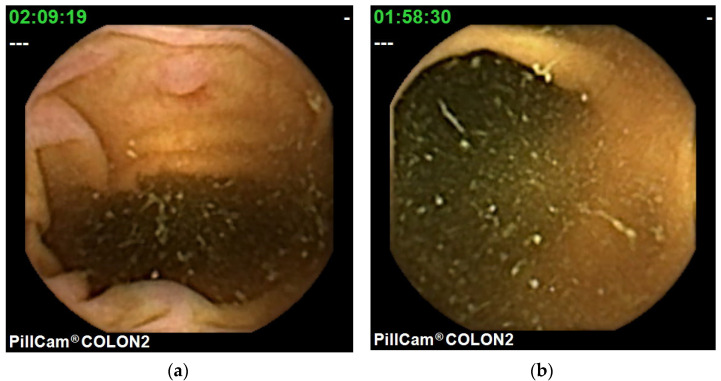
Mucosa visualization in CCE: (**a**) adequate (good), (**b**) inadequate (fair).

A Leighton–Rex scale [87] has been developed for CCE with PillCam COLON. Five colonic segments per video (*n* = 40; 196 segments) were rated separately for their cleanliness by a four-point scale as excellent, poor fair, or good, or by a two-point scale as adequate or inadequate, respectively (Figure 5). The per segment kappa value was 0.754 for the two-point and four-point scales, and 0.619 for the four-point scale, and 0.647 and 0.44, respectively, for the overall assessment of videos.

The Colon Capsule CLEansing Assessment and Report (CC-CLEAR) [70] divided the colon into three segments: right-sided, transverse, and left-sided colon. Each segment was scored according to an estimation of the percentage of visualized mucosa (0, <50%; 1, 50–75%; 2, >75%; 3, >90%). The overall cleansing classification was a sum of each segment score, grading excellent (8–9), good (6–7), and inadequate (0–5). Any segment scoring </=1 resulted in inadequate overall classification. In 58 consecutive CCEs, overall cleansing CC-CLEAR classifications inter-observer agreement of two readers was superior compared to the Leighton–Rex scale (Kendall’s W 0.911 vs. 0.806, respectively; *p* < 0.01).

A computer assisted score based on the red/green ratio of still images of PillCam Colon 2 has been developed for discrimination between adequate and inadequate cleansing but needs further improvement regarding specificity [94]. Similarly, an RGB based Supportive Vector Machine had a 47% agreement with expert classification [95].

## 6. Conclusions

Although a large quantity of studies were performed during the last two decades, evidence is still insufficient in some areas related to optimal preparation for capsule endoscopy. A simplified suggestion for the application of clear liquid diet, lavage, prokinetics, and simethicone is shown in Table 2.

Quality assessment is often based on surrogate parameters for lesion detection rates such as the visibility of mucosa and complete documentation of the target organ within the battery lifetime for small bowel and/or colon.

Lavage can improve the visibility of the SB and the colon. Prolonged recording times and more aggressive boosts in colon capsule endoscopy have increased visibility and completeness rates at the price of a loss of convenience for patients. Hence, future research needs to focus on these issues to keep capsule endoscopy minimally invasive.

## Figures and Tables

**Table 1:** Meta-analyses of studies on lavage before small bowel capsule endoscopy versus clear liquids the day before.

Meta-Analysis (First Author)	Outcome Odds’ Ratio (OR) *(95% Confidence Intervall)*		Intervention (Lavage)
	Adequate Mucosal Visualisation	Diagnostic Yield	
Niv et al., 2008 [18]	↑ 78% vs. 49% (*p* < 0.0001) *(65–88%)* vs. *(35–62%)*	n.a.	PEG, NaP, Simethicon
Rokkas et al., 2009 [19]	↑ OR 2.11 *(1.25–3.57)*	↑ OR 1.81 (*1.21–2.54)*	PEG, NaP
Belsey et al., 2012 [20]	↑ OR 2.3 *(1.46–3.63)*	↑ OR 1.88 *(1.24–2.84)*	PEG, NaP
Kotwal et al., 2015 [21]	↑ OR 3.13 *(1.7–5.75)*	↑ OR 1.68 (*1.16–2.42)*	PEG
	↔	↑ OR 1.77 *(1.18–2.64)*	NaP
Yang et al., 2017 [22]	↑ OR 1.27 *(1.14–1.42)*	↔	PEG
Yung et al., 2017 [23]	↑ OR 1.6 (1.08–2.36)	↔	PEG, NaP, Mg-citrat
Gkolfakis et al., 2018 [24]	↔	↔	PEG, NaP, Mg-citrat

**Table 2:** Simplified recommendations for different capsule endoscopy procedures.

Capsule Endoscopy Procedure	Clear Liquids before CE	Lavage	Prokinetics	Simethicon
Esophageal CE	2 h + 100 mL before CE	No	No	No
Gastric CE	Overnight + 1 L before CE	No	No	Yes
Combined Gastric and Small bowel CE	Overnight + 1 L before CE	Yes	No	Yes
Small bowel CE	Overnight	Yes	Only in delayed gastric transit	Yes
Panintestinal CE	Day before	Yes + boosts	Optional, and in delayed gastric transit	Yes
Colon CE	Day before	Yes + boosts	Optional, and in delayed gastric transit	Optional

## Data Availability

Not applicable.
